# Serum neuron-specific enolase levels are upregulated in patients with acute lymphoblastic leukemia and are predictive of prognosis

**DOI:** 10.18632/oncotarget.10473

**Published:** 2016-07-07

**Authors:** Cheng-cheng Liu, Hua Wang, Jing-hua Wang, Liang Wang, Qi-rong Geng, Xiao-qin Chen, Yue Lu

**Affiliations:** ^1^ Department of Hematologic Oncology, Sun Yat-sen University Cancer Center, Guangzhou, Guangdong, 510060, P.R. China; ^2^ State Key Laboratory of Oncology in South China, Guangzhou, Guangdong, 510060, P.R. China; ^3^ Collaborative Innovation Center for Cancer Medicine, Guangzhou, Guangdong, 510060, P.R. China

**Keywords:** acute lymphoblastic leukemia, neuron-specific enolase, prognostic factor, biomarker, targeted therapy

## Abstract

We explored the relationship between neuron-specific enolase (NSE) levels and the clinical features of acute lymphoblastic leukemia (ALL). Seventy ALL patients and forty-two healthy controls were enrolled in this study, and their serum NSE levels were measured using an electrochemiluminescence assay. The serum NSE concentration was higher in ALL patients than in healthy controls. In ALL patients, the mean serum NSE level declined after complete remission (CR) but increased with relapse. In addition, the mean serum NSE level was lower in the CR group than in the non-CR group. High NSE levels were associated with poorer progression-free and overall survival than low NSE levels. Serum NSE levels closely correlated with several clinical features, including the immunophenotype, risk stratification and serum lactate dehydrogenase levels. Multivariate analysis revealed that high NSE expression was an independent prognostic factor in adult ALL patients. *NSE* mRNA levels were also higher in ALL cell lines and bone marrow mononuclear cells from ALL patients than in control cells. These results suggested that NSE could be a clinical prognostic factor and a potential therapeutic target in ALL.

## INTRODUCTION

Acute lymphoblastic leukemia (ALL) is a malignant disease of the bone marrow in which early lymphoid precursors proliferate and take the place of normal hematopoietic cells in the bone marrow [1, 2]. ALL mostly occurs in children, and the five-year event-free survival rate is greater than 80% in patients who receive standard protocol treatment [3]. However, in adults, ALL is usually associated with a worse prognosis. Although intensified chemotherapy protocols may result in 70-90% remission in adult ALL patients, the long-term survival rate is extremely low, around 40% [4–6]. Consequently, the management of ALL is a challenge for both patients and clinicians. When a therapeutic plan is designed for adults with ALL, several clinical and genetic factors must be considered, such as age, white blood cell (WBC) count, minimal residual disease, and genetic features [4, 7, 8]. ALL is a disorder with clinical and biological heterogeneity. Further improvement in the outcome of ALL therapy will require the development of novel, targeted therapies with low toxicity. Additional biological markers remain to be discovered and will be required in order to optimize the classification and treatment of patients.

Neuron-specific enolase (NSE) is the γ–γ or γ–δ dimer isoenzyme of enolase, an acidic protease that promotes the conversion of β-glycerophosphate into dihydroxyacetone phosphate during glycolysis, and is mainly found in mature neurons and cells of neuronal origin [9, 10]. Studies of NSE as a tumor marker have primarily focused on patients with small cell lung cancer and neuroblastoma [11–13]. NSE is not only a useful marker for disease aggressiveness, but also a prognostic factor. Elevated serum NSE expression has been found in 35% of non-neuroendocrine tumors, such as non-small cell lung carcinoma, breast cancer, lymphoma, and multiple myeloma [14–17]. We previously detected positive expression of serum NSE in 54% of patients with diffuse large B-cell lymphoma, and serum NSE expression closely correlated with Ann-Arbor stages, performance status, International Prognostic Index scores, and serum lactate dehydrogenase (LDH) levels. Serum NSE levels significantly declined in patients who responded to chemotherapy [18, 19]. However, no reports have indicated the whether serum NSE levels influence the clinical features and prognosis of ALL. In this study, we analyzed the serum NSE levels of 70 adult patients with newly diagnosed ALL and investigated their correlation with clinical outcomes. We also investigated the expression of *NSE* in ALL cell lines and bone marrow mononuclear cells (BM-MCs) from ALL patients.

## RESULTS

### Patients' characteristics and correlation with NSE levels

**Figure 1:**
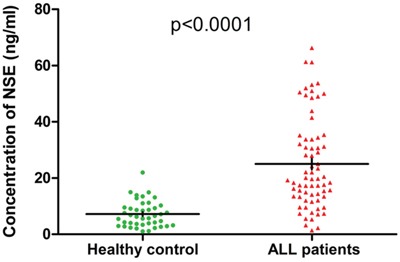
Serum NSE levels in acute lymphoblastic leukemia patients and healthy controls The mean concentration of NSE for 70 acute lymphoblastic leukemia patients was 25.01 ng/mL, significantly higher than that of the 42 healthy controls (7.187 ng/mL, *P*< 0.0001).

The clinical baseline characteristics (including age, gender, immunophenotype, risk stratification and so on) for patients are shown in Table 1. The ratio of males to females was 1.8:1, and the median age of the entire cohort was 28 years (range: 15-72). Forty percent of the patients were at high risk. Forty-two healthy controls (24 males, 18 females; age range 16-50 years) who were healthy blood donors with normal laboratory tests and no history of malignancies were recruited. The mean serum NSE concentration for ALL patients was 25.01 ng/mL, significantly higher than that of healthy controls (7.187 ng/mL, *P* < 0.0001, Figure 1). Serum NSE values closely correlated with the immunophenotype, risk stratification, and serum LDH levels (*P*< 0.05), but were not related to age, gender, or the *BCR/ABL1* gene (*P*>0.05) (Table 1). The serum NSE levels of patients at high risk were significantly higher than those at standard risk (*P* = 0.001). The NSE levels of patients with the T immunophenotype or elevated LDH levels were much higher than those of patients with the B immunophenotype (*P* = 0.002) or normal LDH levels (*P* = 0.013), respectively (Table 1).

**Table 1:** Patients' characteristics and serum NSE level

	N=70(%)	Mean serum NSE level (ng/ml)	P value
Age, years			
15~39	59(84.3%)	26.16±2.13	0.173
≥40	11(15.7%)	18.84±4.57	
Gender			
Male	45(64.3%)	25.43±2.40	0.775
Female	25(35.7%)	24.26±3.37	
Immunophenotype			
B	44(62.9%)	20.52±2.31	0.002*
T	26(37.1%)	32.61±2.98	
BCR/ABL1			
Absent	62(88.6%)	24.15±2.01	0.122
Present	8(11.4%)	31.73±6.90	
WBC count(×10^9^)			
<30	42(60.0%)	22.24±2.35	0.082
≥30	28(40.0%)	29.16±3.25	
Risk stratification			
Standard risk	42(60.0%)	19.14±1.67	0.001*
High risk	28(40.0%)	33.81±3.61	
Serum LDH level			
Normal	12(17.1%)	14.44±4.90	0.013*
>normal	58(82.9%)	27.20±2.02	
Treatment response			
CR	58(82.9%)	22.96±1.97	0.020*
Non-CR	12(17.1%)	34.91±5.53	

Abbreviations: WBC, white blood cell; NSE, neuron specific enolase; LDH, lactate dehydrogenase; CR, complete remission.

### Treatment outcomes and correlation with NSE levels

**Figure 2:**
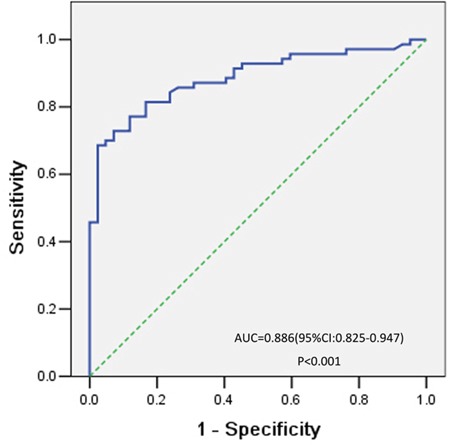
ROC curve analysis of the optimal cutoff point for the serum NSE concentration The most discriminative cutoff value for NSE was 15.2 ng/mL, with an AUC value of 0.886 (*P* < 0.001). The sensitivity and specificity were 68.6% and 97.6%, respectively.

**Figure 3:**
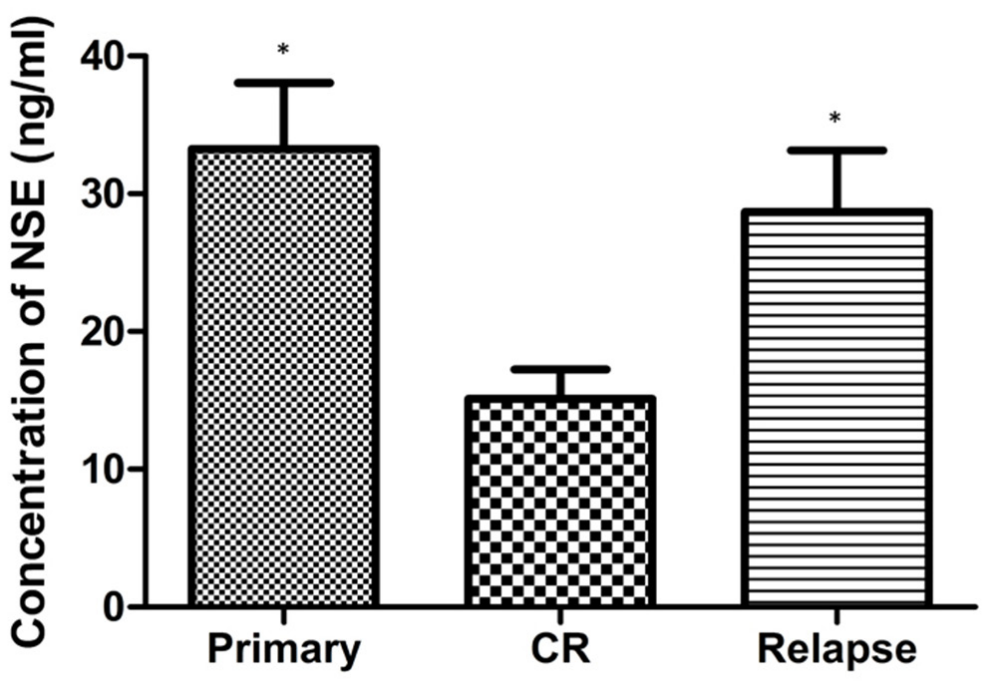
Serum NSE levels of patients at the time of diagnosis, CR, and relapse *P<0.05, compared with CR.

After induction therapy, all patients were eligible for response evaluation. Fifty-eight patients achieved CR. The mean serum NSE level was lower in the CR group than in the non-CR group (P = 0.020) (Table 1). As shown in Figure 2, the best cutoff value defined by a receiver operating characteristic (ROC) curve for serum NSE was 15.2 ng/mL, with an area under the curve (AUC) value of 0.886 [95% confidence interval (CI) 0.825–0.947, *P*< 0.001]. Based on this cutoff value, 48 patients with ALL (68.6%) were classified into the high-NSE-level or elevated NSE group (>15.2 ng/mL), while the remaining 22 patients (31.4%) were classified into the low-NSE-level or normal NSE group (≤15.2 ng/mL). The WBC count, percentage of bone marrow blast and LDH levels were all higher in the elevated NSE group than in the normal NSE group (Table 2). At the time of diagnosis, CR, and relapse, we analyzed the serum NSE levels of patients who relapsed after CR. The mean serum NSE level declined significantly after induction therapy (before treatment: 33.24 ± 4.79 ng/mL, after complete remission: 15.11 ± 2.15 ng/mL; n = 15, *P* = 0.013) but increased with relapse (28.69 ± 4.46 ng/mL) (Figure 3).

**Table 2:** Correlation between NSE expression and clinical features

Parameters	High-NSE	Low-NSE	P value
**WBC(×10^9^)** (mean±SD)	115.43±31.03	20.57±6.05	0.040*
**HB(g/L)** (mean±SD)	88.84±4.27	87.96±7.21	0.918
**PLT(×10^9^)** (mean±SD)	78.22±12.64	108.29±19.29	0.200
**BM blast(%)** (mean±SD)	74.45±3.76	55.91±6.45	0.011*
**LDH(U/L)** (mean±SD)	1157.48±167.45	347.44±51.38	0.002*

Abbreviations: NSE, neuron specific enolase; WBC, white blood cell; HB, hemoglobin; PLT, platelet; BM, bone marrow; LDH, lactate dehydrogenase.

### Correlation of NSE expression with survival

**Figure 4:**
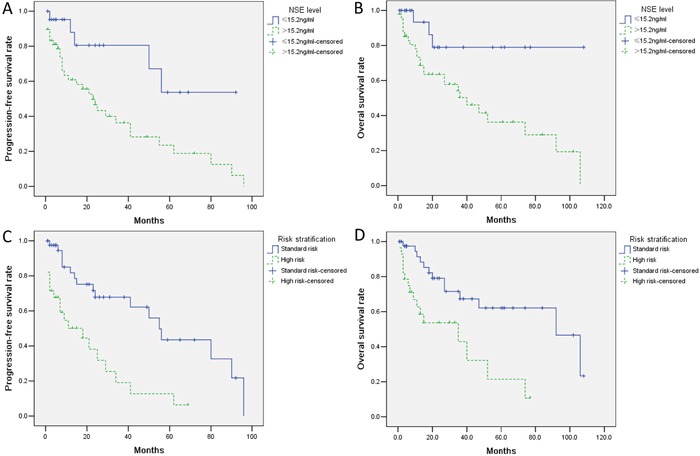
Kaplan-Meier survival analysis for all patients with acute lymphoblastic leukemia Progression-free survival (PFS) and overall survival (OS) were significantly longer in low-NSE patients (≤15.2ng/mL, **A, B.**) and standard-risk patients **C, D.** than in high-NSE and high-risk patients, respectively.

The median follow-up time was 36 months (range 9-108 months). The five-year progression-free survival (PFS) and overall survival (OS) rates were both significantly lower in the high-NSE-level group than in the low-NSE-level group (*P*<0.05; Figure 4A, 4B). The five-year PFS and OS rates were higher in standard-risk patients than in high-risk patients (*P*<0.05; Figure 4C, 4D). Differences in survival were also observed between the patients with high serum NSE level and those with low serum NSE level in B-ALL(*P*<0.05; Supplementary Figure S1A, S1B), Ph-negative ALL(*P*<0.05; Supplementary Figure S2A, S2B). Survival did not differ between the patients with high serum NSE level and those with low serum NSE level in T-ALL, that maybe influenced by the limitation of the number of patients (*P*>0.05; Supplementary Figure S1C, S1D). In the Cox regression model, multivariate analysis revealed that the independent prognostic factors for PFS and OS were the serum NSE level and risk stratification (Table 3).

**Table 3:** Multivariate survival analysis in patients with ALL

Parameters	PFS			OS		
	Univariate analysis	Multivariate analysis		Univariate analysis	Multivariate analysis	
	P value	RR(95%CI)	P value	P value	RR(95%CI)	P value
Age	0.026			0.116		
Gender	0.269			0.304		
Immunophenotype	0.553			0.442		
Risk stratification	<0.001	3.831(1.783-8.230)	0.001	0.009	2.952(1.296-6.726)	0.01
Serum LDH level	0.532			0.636		
Serum NSE level	0.023	2.959(1.104-7.934)	0.031	0.022	5.375(1.235-23.398)	0.025

Abbreviations: NSE, neuron specific enolase; LDH, lactate dehydrogenase; PFS, progression-free survival; OS, overall survival; RR, relative risk; CI, confidence interval.

### Expression of *NSE* in ALL cell lines and BM-MCs from ALL patients

**Figure 5:**
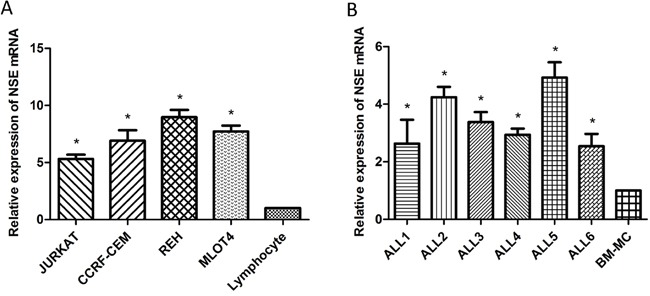
*NSE* mRNA expression in cell lines and bone marrow cells by real-time PCR analysis **A.** The *NSE* levels were significantly higher in all the ALL cell lines than in normal peripheral blood lymphocytes. *P<0.05, compared with normal peripheral blood lymphocytes. **B.** The *NSE* levels were significantly higher in the bone marrow mononuclear cells (BM-MCs) of ALL patients than in healthy controls. *P<0.05, compared with BM-MCs of healthy controls.

We investigated *NSE* mRNA expression in cell lines and bone marrow samples. As shown in Figure 5A, the *NSE* mRNA levels of four kinds of ALL cells JURKAT(fold change=5.31, *P*<0.05), CCRF-CEM(fold change=6.91, *P*<0.05), REH(fold change=8.97, *P*<0.05) and MOLT-4(fold change=7.72, *P*<0.05) were all higher than those of normal peripheral blood lymphocytes, and the level in REH cells was the highest. Furthermore, the *NSE* mRNA levels of the BM-MCs of six ALL patients were also significantly higher than those of healthy controls (Figure 5B).

## DISCUSSION

ALL is the most common type of acute leukemia, accounting for 15–20% of adult acute leukemia cases. Although more than 80% of adult ALL patients can achieve CR, the majority of patients with higher risk features at diagnosis eventually relapse, so the long-term survival rate is only 20-40% for patients younger than 60 years, and less than 10% for those older than 60 years [20]. Several risk factors are recognized as being independently associated with poor outcomes in adult ALL, including older age, high WBC count, and cytogenetic abnormalities such as Philadelphia chromosome-positivity, MLL gene amplification, complex karyotypes, and others. Ph-positivity confers a uniformly poor prognosis to patients treated with standard chemotherapy [21, 22]. It is increasingly apparent that the identification of tumor markers is valuable for the diagnosis and treatment of various diseases. Thus, the discovery of additional biological markers may guide the development of appropriate treatments and increase the accuracy of prognostic evaluations for ALL patients.

NSE is considered to be a tumor marker, and its diagnostic value has gained interest among clinicians [23]. In recent years, NSE has been found in a variety of tumors, including malignant lymphomas. We previously found that the treatment response and prognosis differed between normal- and elevated-NSE groups with Non-Hodgkin's lymphoma [18, 19]. However, the clinical significance of NSE in ALL, such as the correlation of serum NSE levels with disease progression or prognosis, was not mentioned in previous reports.

In this study, serum NSE levels were elevated in 68.6% of patients with newly diagnosed ALL. The frequency of NSE-positivity in ALL was similar to that in small cell lung cancer (about 70%) and higher than that previously reported in Hodgkin's lymphoma (6.5%) and Non-Hodgkin's lymphoma (17.2%) [14, 24]. We also found that the mRNA expression of *NSE* was higher in ALL cell lines and BM-MCs from ALL patients and than in normal lymphocytes. These findings indicate that tumor cells are at least partially responsible for producing and secreting NSE into the serum.

The reason that the production of NSE is accelerated in ALL is not clear. Mohammad et al., who used a stimulated B-cell line of diffuse large cell lymphoma cells, demonstrated that the excessive metabolism of lymphoma cells produces enolase. Normal B-lymphocytes in the late stage of differentiation were found to be NSE-positive by immunohistochemistry [25]. However, we found that B-lymphocytes and T-lymphocytes in ALL were all NSE-positive. High mRNA expression of *NSE* led to elevated serum NSE levels in patients with ALL. It was reported that when NSE expression was increased in glioblastoma cell lines, the protein and phosphoprotein levels in the PI3K/Akt, MAPK/ERK and anti-apoptotic signaling pathways increased significantly. Thus, elevated serum levels of NSE in patients may be a marker of disease progression. Although the exact mechanism of NSE production in ALL cells remains unclear, the present study demonstrates that positive serum NSE expression is an important prognostic factor in ALL.

We evaluated patients who responded to chemotherapy, and found that serum NSE levels declined after CR but increased with relapse. Thus, NSE may reflect the disease progression of patients. The tumor burden was alleviated when NSE levels decreased to some extent after induction therapy. Elevated NSE levels could be due to the acceleration of the cell cycle and enhancement of glycolysis during tumor cell growth. NSE is an acidic protease that promotes the conversion of β-glycerophosphate into dihydroxyacetone phosphate during glycolysis. The upregulation of intracellular NSE in tumor cells may lead to increased release of NSE into the blood. After chemotherapy, the number of tumor cells decreases and cell division slows, so the NSE level can decline to a normal level. Our findings support the regular monitoring of serum NSE levels during the management of ALL patients. We will continue to expand the total sample size in order to study the correlation between NSE levels and disease progression.

NSE has become an important indicator for cancer patients. For example, in lung cancer, NSE evaluation can aid in the diagnosis of pathological type, stage, metastasis, recurrence, and prognosis. In this study, we found that serum NSE values positively correlated with clinical factors of ALL, such as immunophenotype, risk stratification and LDH levels. The serum NSE levels paralleled the serum LDH levels, suggesting that serum NSE might reflect the disease status and predict the prognosis. Kaplan–Meier analysis revealed a significant association of high NSE levels with inferior PFS and OS. Multivariate analysis revealed that the serum NSE level was an independent prognostic factor in adult patients with ALL. Although our findings suggest that the NSE level influences ALL prognosis, our conclusions are limited due to the retrospective nature of this study. To obtain stronger evidence, future studies should be conducted among larger samples of patients to verify the prognostic relevance of serum NSE levels. Additionally, laboratory work needs to be done to clarify the mechanism of NSE production in ALL.

In conclusion, this is the first study to confirm the association of NSE levels with several clinical features of ALL. High serum NSE levels independently predict poor prognosis in adult ALL patients. As the serum NSE level can be measured easily in clinical practice, it may be an important monitoring index of disease development. Effective prognostic factors such as NSE should be used to classify patients with ALL, and treatments such as hematopoietic stem cell transplantation and more aggressive chemotherapies should be administered to patients with unfavorable prognoses. Although the exact mechanism of NSE production in ALL remains unclear, these findings suggest that the serum NSE level could be used as a new prognostic indicator and be targeted in therapeutic strategies.

## MATERIALS AND METHODS

### Ethics statement

This study was approved by the Institutional Review Board of Sun Yat-sen University Cancer Center, and written informed consent was obtained from every healthy volunteer and patient prior to treatment. Additionally, this study was conducted in accordance with the Declaration of Helsinki.

### Patients

This study used a retrospective cohort study design. Seventy adult patients diagnosed with ALL were enrolled between January 2002 and December 2014 at the Department of Hematologic Oncology, Cancer Center, Sun Yat-Sen University. The inclusion criteria for this study were as follows: (1) confirmed diagnosis of ALL according to the French-American-British (FAB) classification system, based on clinical features, bone marrow cytology, flow cytometric evaluation, and cytogenetic detection [1]; (2) availability of serum at diagnosis; (3) complete clinical information and follow-up data. Patients with extramedullary disease and those who received allogenic stem cell transplantation were excluded. Forty-two healthy blood donors were recruited as controls. Clinical indicators were routinely examined. Data were obtained from the patients' records. Approval to review, analyze, and publish the data in this study was given by the Sun Yat-sen University Cancer Center Research Ethics Board. The patients' blood samples were collected before and after the treatment.

### Treatment strategies and response evaluation

Thirty-seven patients in this study were treated with the JALSG ALL 87 protocol, and 33 patients were treated with the MRC UKALLXII/ECOG E2993 protocol (from a joint international study initiated by the Medical Research Council in the UK and the Eastern Cooperative Oncology Group in the US in 1993). Details of the treatment protocols have been published previously [26–28]. After induction therapy and intensification therapy, most of patients received maintenance therapy, either because bone marrow donors were not available or because the patients could not afford to pay the expensive medical fees for transplantation. In our study, ten ALL patients received allogeneic transplantation. For outcome analyses, we evaluated whether patients reached CR, which was defined as having less than 5% blast cells in the bone marrow and showing evidence of normal maturation of other marrow elements after induction chemotherapy [2]. Patients with other treatment responses, including partial remission, non-remission, and early death, were assigned to the non-CR group. Relapse was defined by the appearance of more than 5% lymphoblast in a single bone marrow aspirate or leukemic cell infiltration in extramedullary organs. PFS was established as the time between the date of diagnosis and the date of disease progression or death, and was determined at the last follow-up visit. OS was defined as the interval between the date of diagnosis and death or last follow-up.

### Serum NSE measurement

Venous blood was obtained from patients immediately before and after induction therapy. The electrochemiluminescence assay kit (Roche Diagnostics, Indianapolis, IN, USA) was used to measure serum NSE values. This method employs an NSE-specific monoclonal antibody labeled with a ruthenium complex, which emits light when activated. Reactions and quantification were performed with fully automatized equipment (Roche Diagnostics Corporation). Internal software and controls provided by the manufacturer were used to control the quality of the assay. All samples were analyzed in duplicate, experiments were repeated three times, and the results are presented as the means ± standard deviations.

### Real-time quantitative RT-PCR

BM-MCs were enriched by Ficoll–Hypaque gradient centrifugation and preserved in liquid nitrogen. The *NSE* mRNA levels in BM-MCs and ALL cell lines were detected by a real-time quantitative Reverse Transcription Polymerase Chain Reaction (RT-PCR) according to the manufacturer's instructions. The primer sequences used for RT-PCR were as follows - *NSE*: forward 5′-TATGGATGTGGCTGCCTCTG-3′, reverse 5′-TGGTGATTGGTATGGATGTGG-3′; *GAPDH*: forward 5′-CCATGGAGAAGGCTGGGG-3′, reverse 5′-CAAAGTTGTCATGGATGACC-3′. Real-time quantitative RT-PCR was performed for each sample in duplicate with *GAPDH* as a housekeeping gene. The comparative cycle threshold (Ct) method was used to determine the relative expression of *NSE*. Each sample was tested in triplicate.

### Statistical analysis

All statistical analyses were performed with SPSS software (version 13.0, SPSS Inc., Chicago, IL, USA). ROC curve analysis was performed to determine the best cutoff value for the NSE concentration. In this ROC curve, the point with the maximum sensitivity and specificity was selected as the cutoff value. Correlations between serum NSE levels and clinical features were evaluated with the Chi-squared test for categorical data and the nonparametric Mann–Whitney U-test for continuous variables. PFS and OS were estimated by the Kaplan–Meier method and were compared between groups in a log-rank test. A Cox regression model was used for multivariate analysis to define the prognostic significance of the selected variables, including NSE levels. The results of comparative tests were considered significantly different if the two-sided p-value was less than 0.05.

## SUPPLEMENTARY FIGURES AND TABLES


