# Supplementary material for: Serum neuron-specific enolase levels are upregulated in patients with acute lymphoblastic leukemia and are predictive of prognosis
Source: Oncotarget. 2016 Jul 7;7(34):55181–90. doi: 10.18632/oncotarget.10473 (PMC5342410; doi:10.18632/oncotarget.10473)
Supplement: Supplementary file 1 [file oncotarget-07-55181-s001.pdf]

Serum neuron-specific enolase levels are upregulated in patients with acute lymphoblastic leukemia and are predictive of prognosis

SUPPLEMENTARY FIGURES AND TABLES

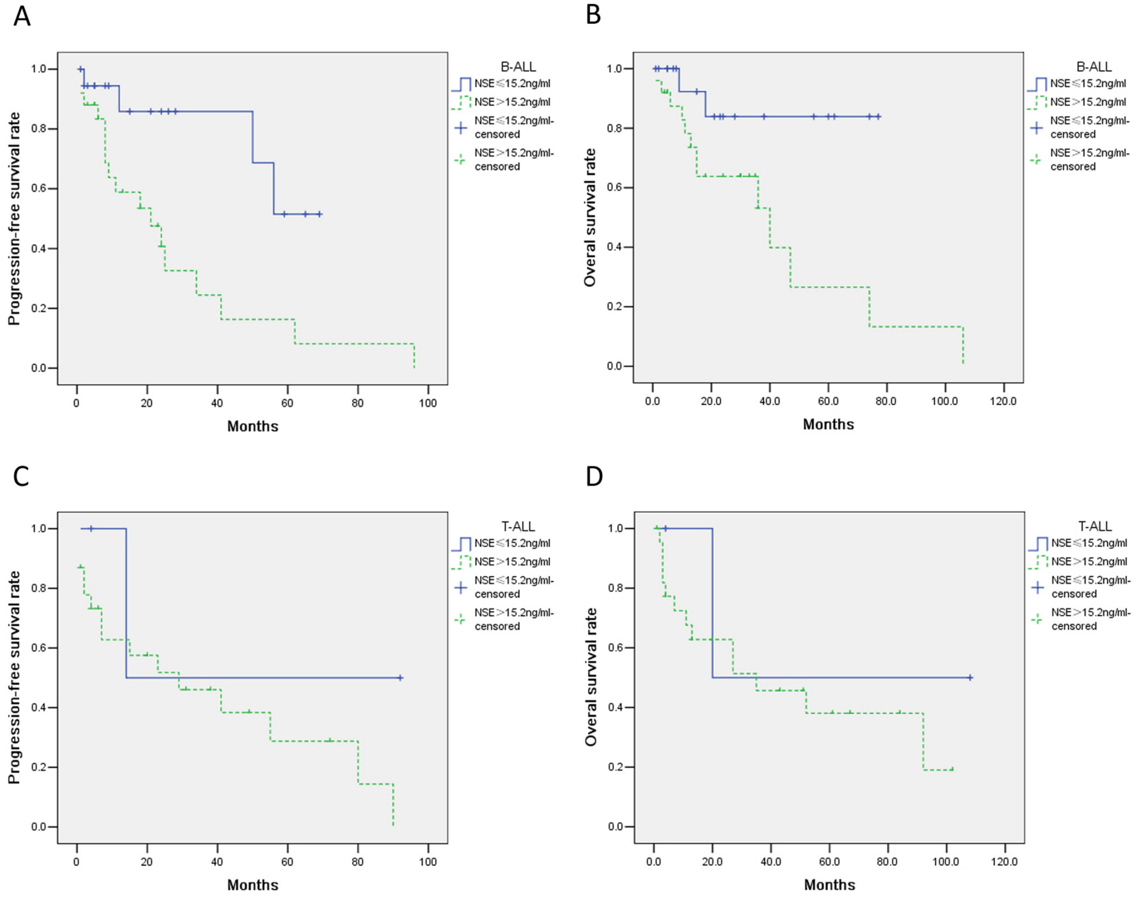

Supplementary Figure S1: Survival outcomes of B-ALL and T-ALL. patients based on serum NSE levels. Progression-free survival (PFS) and overall survival (OS) according to serum NSE level in B-ALL **A, B.** Progression-free survival (PFS) and overall survival (OS) according to serum NSE level in T-ALL **C, D.**

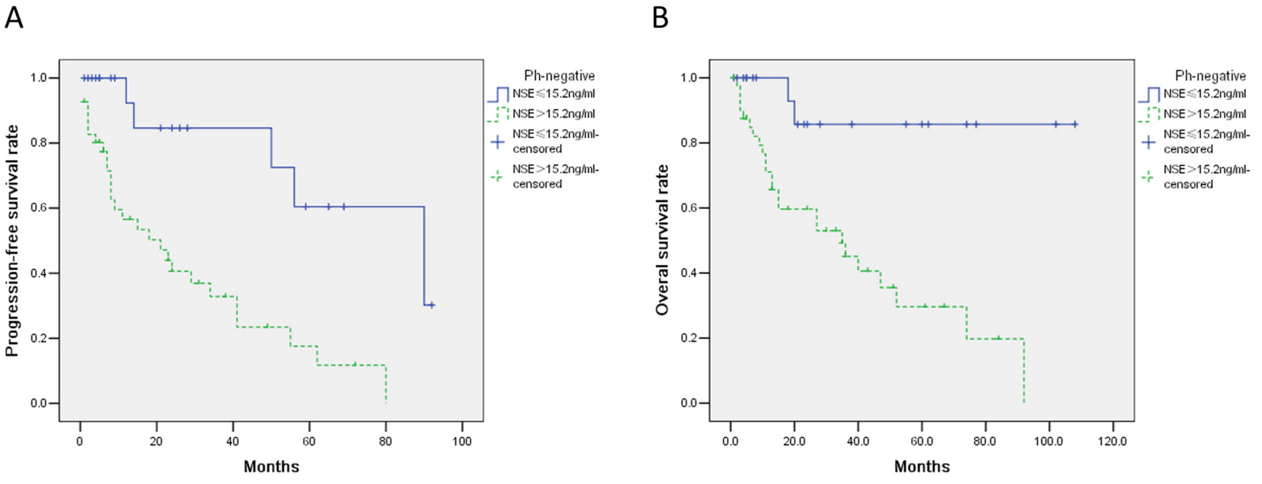

Supplementary Figure S2: Survival outcomes of Ph-negative ALL based on serum NSE levels. A. Progression-free survival (PFS) according to serum NSE level in Ph-negative -ALL. **B.** overall survival (OS) according to serum NSE level in Ph-negative ALL.
